# Microwave birefringent metamaterials for polarization conversion based on spoof surface plasmon polariton modes

**DOI:** 10.1038/srep34518

**Published:** 2016-10-04

**Authors:** Yongfeng Li, Jieqiu Zhang, Hua Ma, Jiafu Wang, Yongqiang Pang, Dayi Feng, Zhuo Xu, Shaobo Qu

**Affiliations:** 1 College of Science, Air Force Engineering University, Xi,an 710051, People’s Republic of China; 2 Electronic Materials Research Laboratory, Key Laboratory of Ministry of Education, Xi’an Jiao-tong University, Xi’an 710049, People’s Republic of China

## Abstract

We propose the design of wideband birefringent metamaterials based on spoof surface plasmon polaritons (SSPPs). Spatial *k*-dispersion design of SSPP modes in metamaterials is adopted to achieve high-efficiency transmission of electromagnetic waves through the metamaterial layer. By anisotropic design, the transmission phase accumulation in metamaterials can be independently modulated for *x*- and *y*-polarized components of incident waves. Since the dispersion curve of SSPPs is nonlinear, frequency-dependent phase differences can be obtained between the two orthogonal components of transmitted waves. As an example, we demonstrate a microwave birefringent metamaterials composed of fishbone structures. The full-polarization-state conversions on the zero-longitude line of Poincaré sphere can be fulfilled twice in 6–20 GHz for both linearly polarized (LP) and circularly polarized (CP) waves incidence. Besides, at a given frequency, the full-polarization-state conversion can be achieved by changing the polarization angle of the incident LP waves. Both the simulation and experiment results verify the high-efficiency polarization conversion functions of the birefringent metamaterial, including circular-to-circular, circular-to-linear(linear-to-circular), linear-to-linear polarization conversions.

The traditional birefringent materials mainly refer to birefringent crystals^1^,^2^,^3^ such as calcite and mica. They are always cut with the optic axis oriented such that there is a large difference in the refractive index along orthogonal axes, leading to a strong birefringence. For light incidence upon the birefringent crystals, the polarization is decomposed along the two axes. In the propagation process, the components along the two axes will encounter different refraction index coefficients. Thus, the phase difference between the components depends on the thickness of the crystals. Metamaterials^4^,^5^ are composed of sub-wavelength elements designed to offer specific electromagnetic responses usually not found in nature. The electromagnetic responses just depend on the geometric of the elements. Therefore, anisotropic metamaterials can be achieved with ease. This anisotropy opens the door for birefringent metamaterials^6^,^7^,^8^,^9^. In comparison with conventional birefringent materials, birefringent metamaterials usually have smaller volumes and are more suitable for compact optical components.

Polarization is an important characteristic of electromagnetic waves due to the inherent polarization sensitivity of materials, especially in the visible spectrum^1^,^2^. Conventional polarization conversions are always realized using the wave-plate, for which strong birefringence and a large phase delay are highly desirable. Therefore, birefringent materials such as crystalline solids and liquid crystals^3^ are always employed. The ferrite phase shifter and multi-layered grating polarizer are usually employed in the microwave frequency band. However, the large thickness and the narrow bandwidth prevent them from being integrated into the micro-optical systems. Currently, polarization conversions can be achieved by the anisotropic or chiral metamaterials^10^,^11^, yet still with thickness limitations and narrow bandwidth. Until recent years, metasurfaces^12^,^13^,^14^,^15^,^16^,^17^,^18^,^19^,^20^,^21^,^22^,^23^,^24^,^25^,^26^ provide another way for polarization manipulation. But it is difficult to simultaneously achieve an abrupt phase shift and a high-efficiency transmittivity in a wide frequency band for transmissive metasurface.

In this paper, spoof surface plasmon polaritons (SSPPs)^27^,^28^ mediated by the metallic blade structure is studied by the dispersion relationship. The transmission based on the SSPP coupling is proposed and improved by spatially varied design of the propagation constant along the propagation direction using a fishbone structure. The principle of metamaterials with arbitrary refractive index coefficient achieved based on SSPP mode coupling is proposed and analyzed. The dispersion of the equivalent refractive index coefficient can be modulated via manipulating the dispersion of the mediated SSPP with much freedom. Based on this mechanism, a microwave birefringent metamaterial is achieved via designing the refractive index coefficients independently in two orthogonal directions, where the equivalent refractive index coefficient in one direction is strong dispersive, and weak dispersive in the other direction. By using this birefringent metamaterial, multi-functional polarization conversion has been achieved by a nonlinearly dispersive refractive index coefficient difference between the two orthogonal directions. Both the simulations and experiments verified the high-efficiency circular-to-circular (CTC), circular-to-linear (CTL) (linear-to-circular, LTC), and linear-to-linear (LTL) polarization conversion transmissions, respectively, at different frequency ranges.

## Result

### Microwave birefringent metamaterial

**Figure 1:**
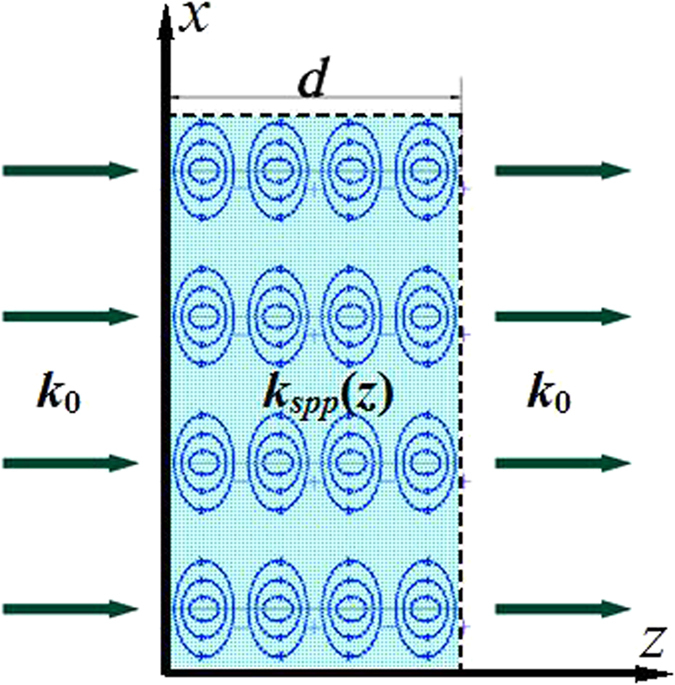
Principle of the metamaterial with an arbitrary refractive index coefficient.

Metamaterials with an arbitrary refractive index coefficient can be achieved based on the SSPP modes as shown in Fig. 1. The thickness of the metamaterial is *d*. The incidence wave is coupled into the SSPP in the constructed metamaterial. *k*_*spp*_(*z*) is the spatially dispersive propagation constant of the coupled SSPP. The equivalent refractive index coefficient *n* can be derived by


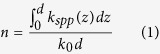


where *k*_0_ is the propagation constant of the wave in free space. The equivalent refractive index coefficient of the achieved metamaterial can be manipulated by modulating the propagation constant of the coupled SSPP with great freedom. More important, the spatial dispersion of the coupled SSPPs for *y-* and *x*-polarized wave incidence can be independently designed, respectively.

**Figure 2:**
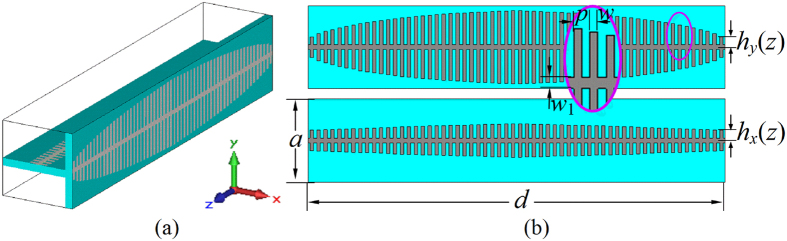
Schematic pictures of the unit cell of the designed microwave birefringent metamaterial. (**a**) Perspective view of the unit cell of the microwave birefringent metamaterial, (**b**) Front views of the fishbone structures in *yoz* and *xoz* planes.

By means of this principle, a microwave birefringent metamaterial based on SSPPs is designed, as shown in Fig. 2. The unit cell of the microwave birefringent metamaterial is composed of two different metallic fishbone structures placed orthogonally. Figure 2(a) gives the perspective view of the unit cell, and Fig. 2(b) the front view of the two metallic fishbone structures, which are employed to couple and guide the SSPPs. The fishbone structure is designed by modulating the metallic blade length *h* according to the spatial dispersive propagation constant of the mediated SSPP *k*_spp_(*z*). With the increased spatial coordinate *z*, the propagation constant *k*_spp_(*z*) firstly increases, and then decreases. This particular design can realize high-efficiency SSPP coupling via matching propagation constant of the SSPP and free space wave.

### SSPP mediated by metallic blade structure

**Figure 3:**
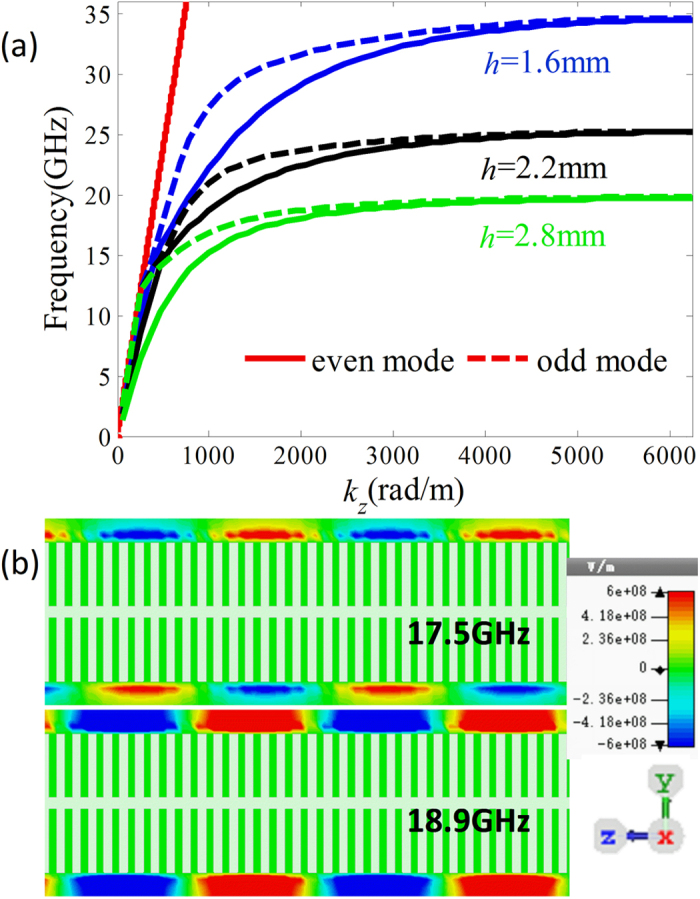
SSPP mediated by metallic blade structures. (**a**) Dispersion relations of the SSPP supported by the blade structures with different blade length *h* = 1.6 mm, 2.2 mm, and 2.8 mm. (**b**) Distributions of the electric field component *E*_*x*_ in the *y*o*z* plane for odd and even modes at different frequencies (17.5 and 18.9 GHz) on the blade structure with *h* = 2.2 mm.

As shown in Fig. 2, the metallic blade structure is proposed to mediate the SSPPs at microwave frequencies^28^. The metallic blade structure is etched on the 0.6 mm-thick F4B (*ε*_r_ = 2.65, tan *δ* = 0.001) dielectric substrate. The geometrical parameters are designed to be: *a* = 6 mm, *p* = 0.5 mm, *w* = 0.25 mm, and *w*_1_ = 0.35 mm. The dispersion diagrams of the *y*-polarized waves on this blade structure with different length *h* (1.6 mm, 2.2 mm, and 2.8 mm) are simulated and given in Fig. 3(a). The red solid line is the light line. It can be found that all the dispersion relations lie below the light line. This means that the propagation constant *k*_*z*_ for the *y*-polarized wave on the blade structure is much larger than which for the wave in free space. Thus the *y*-polarized wave propagating on the blade structure can be considered as the SSPP in microwave frequency regime, which are confined and enhanced within the sub-wavelength regions. This can be demonstrated by the electric field modes given in Fig. 3(b). It is observed that electric fields of the SSPP are highly confined on the blade structure. The wavelength of the SSPPs observed from the distributions of the electric fields is highly reduced compared with the wavelength in free space. Moreover, the dispersion curves include two branches corresponding to the even and odd modes, respectively, and the asymptotic frequencies of two modes are quite close to each other. This also can be demonstrated by the simulated electric filed mode component *E*_*x*_ at 17.5 GHz and 18.9 GHz, respectively, corresponding to odd and even mode. Additionally, the propagation mode will be cut off as the frequency is greater than the asymptotic frequency. Both the asymptotic frequencies and the propagation constant at a fixed frequency can be easily tailored by changing the blade length *h*. In detail, the propagation constant of the SSPP increases with increasing blade length *h*, and the asymptotic frequency moves toward the lower frequency as the blade length *h* is increased.

### High-efficiency transmission based on SSPP coupling

**Figure 4:**
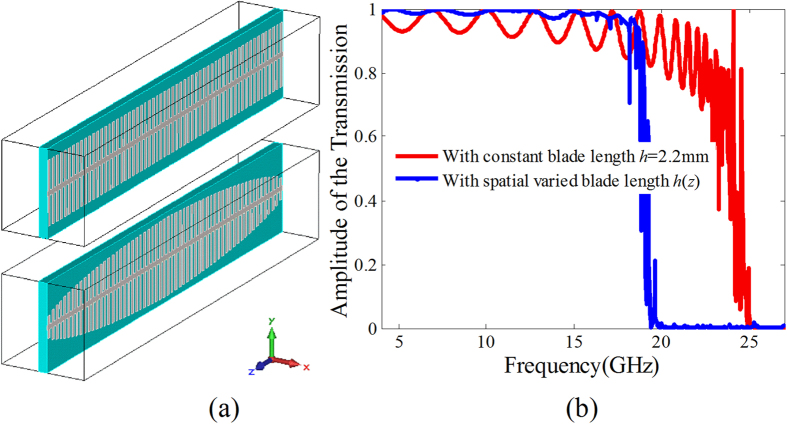
Improvement of the SSPP coupling based transmission by metallic fishbone structure. (**a**) The perspective view of the blade structure with the constant blade length *h* = 2.2 mm and the fishbone structure, (**b**) Comparison of the transmissions for *y*-polarized wave normal incidence onto the blade structures with constant blade length and the fishbone structure.

As for the periodically arranged blade structure consisting of 60 blades with the blade length *h* = 2.2 mm (see the top unit cell in Fig. 4(a)), the simulated amplitude of the transmission coefficient under normally *y*-polarized wave incidence is given by the red line in Fig. 4(b). It is found that the *y-*polarized wave transmits through the periodic blade structure array as the frequency is smaller than 25 GHz, and highly reflected as the frequency *f* > 25 GHz. This is well consistent with the dispersion relationship of the SSPP given in Fig. 3(a), in which the SSPP mode is cut off as the frequency *f* > 25 GHz. Therefore, the transmission under *y*-polarized wave incidence is attributed to the SSPP coupling on the blade structure. However, the transmission is comparatively low especially at the higher frequency region. The transmission is enormously reduced less than 0.8 as the frequency *f* > 20 GHz. This is owing to the mismatching of the propagation constant between the waves in free space and the SSPP on the blade structure. To enhance the transmission, the propagation constant matching in the propagation direction is improved by a spatially varied propagation constant design along the propagation direction using fishbone structure with spatially varied blade length *h*(z). In detail, we designed the fishbone structure, on which the SSPP has a linear propagation constant distribution. The propagation constant of the SSPP on the blade near the free space have the smallest value. To verify the transmission improvement of the fishbone structure, we designed a fishbone structure with the total phase accumulation of the guided SSPP equal to that with constant blade length *h* = 2.2 mm as shown in Fig. 4(a). The simulated transmission in the frequency range 5–20 GHz is given in Fig. 4(b) using the blue dashed line. Obviously, the transmission of the fishbone structure is highly improved.

### Equivalent refractive index coefficient

**Figure 5:**
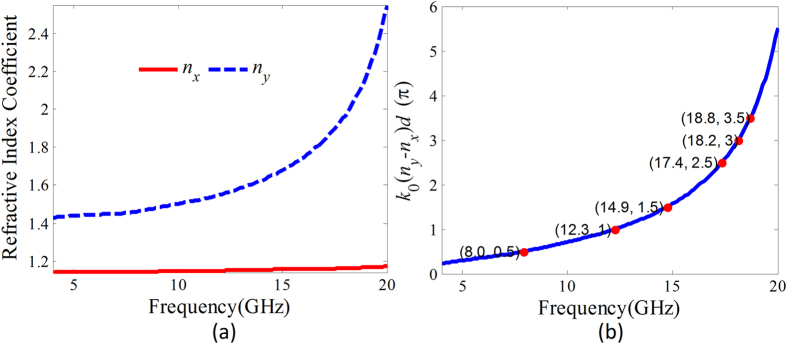
Equivalent refractive index coefficients derived from the dispersion of the mediated SSPP and the phase difference between the *x*- and *y*-polarized components. (**a**) The equivalent refractive index coefficients for *x-* and *y-*polarized wave normal incidence. (**b**) The phase difference between the transmissions for *y-* and *x-*polarized wave incidence calculated from the derived refractive index coefficients *n*_*y*_ and *n*_*x*_.

As for the periodically arranged metallic fishbone structure array, the anti-symmetrical SSPP mode (odd mode) can be efficiently coupled under the electromagnetic wave polarized along the metallic blade normal incidence. This can be understood that the first metallic blades can be approximately considered as a dipole array. In this wise, incident waves can be firstly fed into the metallic fishbone structure by the dipole resonance and then the SSPPs are excited. The field distribution of the dipole resonance is anti-symmetric, and thus the odd-mode SSPPs are coupled on the metallic fishbone structure array. Thus, for the proposed unit cell of the microwave birefringent metamaterial, the anti-symmetrical SSPPs on the metallic fishbone structures in *yoz* and *xoz* plane will be almost independently coupled under *y*- and *x*-polarized wave incidence. According to equation (1), the equivalent refractive index coefficients of the proposed birefringent metamaterial can be derived from the dispersions of the SSPPs on the two metallic fishbone structures. The calculated equivalent refractive index coefficients are given in Fig. 5(a), where *n*_*y*_ and *n*_*x*_ are equivalent refractive index coefficients for *y*- and *x*-polarized wave normal incidence, respectively. We can find that the equivalent refractive coefficient *n*_*x*_ is approximately kept constant due to the weak dispersion of the SSPP on the metallic fishbone structure in *xoz* plane in this frequency region. Contrarily, due to the strong dispersion of the SSPP on the metallic fishbone structure in *yoz* plane, the equivalent refractive index coefficient *n*_*y*_ remarkably increases with increasing frequency. The phase difference between the transmissions for *y-* and *x-*polarized waves incidence calculated according to the derived equivalent refractive index coefficients is given in Fig. 5(b). It is found that this phase difference is frequency dispersive. The phase differences π/2, π, 3π/2, 2π, 5π/2 and 3π are respectively corresponding to 8.0, 12.3, 14.9, 17.4, 18.2 and 18.8 GHz.

**Figure 6:**
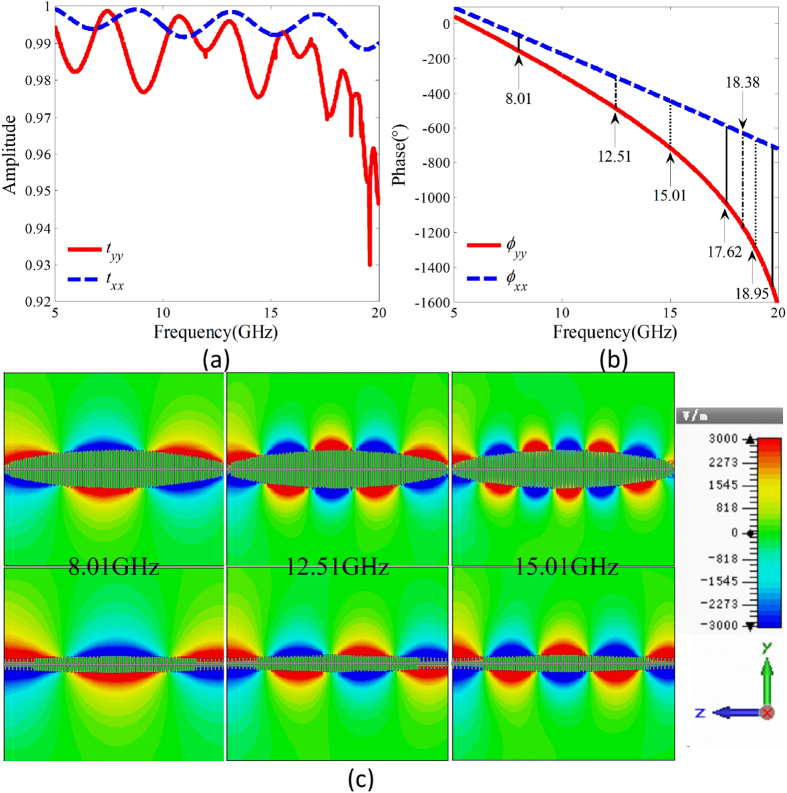
Simulation verification of the designed microwave birefringent metamaterial. (**a**) The amplitudes and (**b**) phases of the simulated co-polarization transmission coefficients under *y*- and *x*-polarized wave normal incidence onto the birefringent metamaterial, respectively. (**c**) Distributions of the electric field *E*_*y*_ of the SSPPs on the fishbone structures in *yoz* plane and the electric field *E*_*x*_ of the SSPPs on the fishbone structure in *xoz* plane at three frequencies of 8.01, 12.51, and 15.01 GHz.

To verify the designed microwave birefringent metamaterial given in Fig. 2, the amplitudes and phases of the transmission coefficients under *y-*and *x-*polarized wave normal incidence are simulated and the results are given in Fig. 6(a,b). It can be found that over a wide frequency range of 5–20 GHz, the amplitudes of the transmission coefficients are all greater than 0.95. The phase difference *φ*_*diff*_ = *φ*_*yy*_ − *φ*_*xx*_ is nonlinearly dispersive due to dispersion characteristic of the SSPPs. The phase differences 90°, 180°, 270°, 450°, 540°, and 630° correspond to the frequencies 8.01, 12.51, 15.01, 17.62, 18.38, and 18.95 GHz, respectively, which are in good accordance with the theoretically predicted frequencies 8.0, 12.3, 14.9, 17.4, 18.2 and 18.8 GHz. The distributions of the electric field *y*-components of the SSPP on the fishbone structure in *yoz* plane and electric field *x*-components of the SSPP on the fishbone structure in *xoz* plane are simulated, respectively, under *y-* and *x*-polarized wave normal incidence. The simulated results at three frequencies 8.01 GHz, 12.5 GHz and 15.01 GHz are given in Fig. 6(c). It can be found that the SSPP are highly confined on the fishbone structures. The phase differences observed from the distributions of the electric fields are 90°, 180° and 270° at the three frequencies, respectively. This reveals a good accordance with the designed values.

### Working principle for polarization conversion

**Figure 7:**
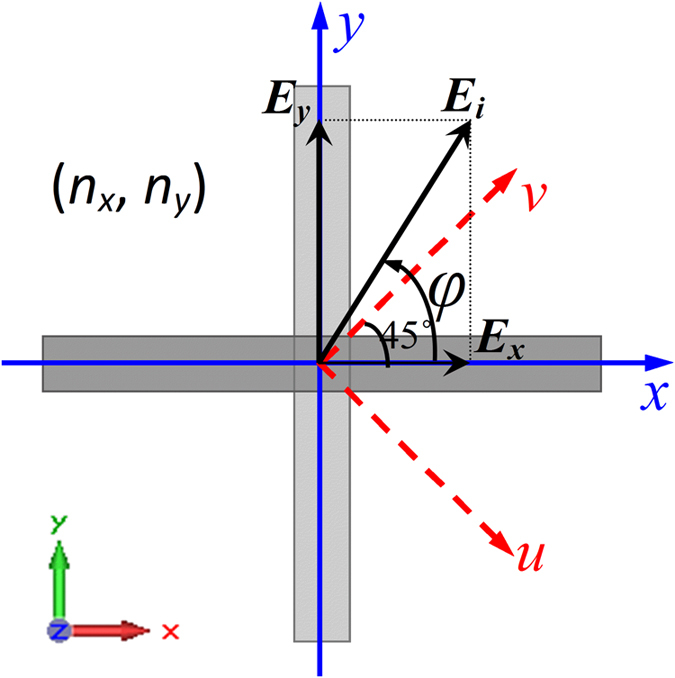
Schematic diagram of the working principle for polarization conversion using the birefringent metamaterial.

As for the designed birefringent metamaterial shown in Fig. 7, electromagnetic waves incident from +z direction. The incident electric field can be expressed as: 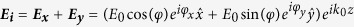. *φ* is the polarization angle of the incidence wave. The *uov* coordinate system is setup at the directions *φ* = ±π/4. If *φ*_*y*_ − *φ*_*x*_ = ±π/2 and *φ* = ±π/4, the incident wave will be circularly polarized (CP), and linearly polarized (LP) as *φ*_*y*_ − *φ*_*x*_ = 0 or *π*. The electric field of the transmitted wave can be derived using the transmission matrix method. That is


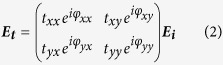


where *t*_*xx(yy)*_ and *t*_*xy(yx)*_ are the amplitudes of the co- and cross-polarization transmission coefficients, *φ*_*xx(yy)*_ and *φ*_*xy(yx)*_ the phases. If the cross-polarization transmissions can be ignored, i.e. *t*_*xy*_ = *t*_*xy*_ = 0, the transmitted electric field can be expressed as:


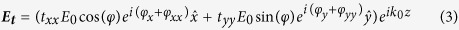


Supposing that all the amplitudes of the co-polarization transmissions have near unity values, i.e. *t*_*xx*_ = *t*_*yy*_ ≈ 1, the phase accumulations of the co-polarization transmissions *φ*_*yy*=_*n*_*y*_*d* and *φ*_*xx*_ = *n*_*x*_*d*, we can conclude that if (*φ*_*y*_ − *φ*_*x*_) + (*φ*_*yy*_ − *φ*_*xx*_) = ±π/2 and *φ* = ±π/4, the transmitted waves will be CP, and LP if (*φ*_*y*_ − *φ*_*x*_) + (*φ*_*yy*_ − *φ*_*xx*_) = 0, or ±π. In other words, under CP wave incidence, if the phase difference *φ*_*yy*_ − *φ*_*xx*_ = ±π, the transmitted wave will be cross-polarized CP, and LP if *φ*_*yy*_ − *φ*_*xx*_ = ±π/2. Under LP wave incidence, if the *φ*_*yy*_ − *φ*_*xx*_ = ±π, the transmitted wave will be cross-polarized LP, and CP if φ_*yy*_ − φ_*xx*_ = ±π/2 and *φ* = ±π/4.

Accordingly, for the designed birefringent metamaterial, we can conclude that at the frequencies *f* = 8.01, 15.01, 17.62 and 18.95 GHz, the CP waves can be converted into *v*-(*u*-)polarized waves, and the *v*-(*u*-)polarized waves can be converted into CP waves. At the frequencies *f* = 12.51 and 18.38 GHz, the LCP(RCP) incidence waves can be converted into RCP(LCP) waves, and the *v*-(*u*-)polarized waves can be converted into *u*-(*v*-)polarized waves.

### Full-polarization-state conversions achieved by varying the frequency

**Figure 8:**
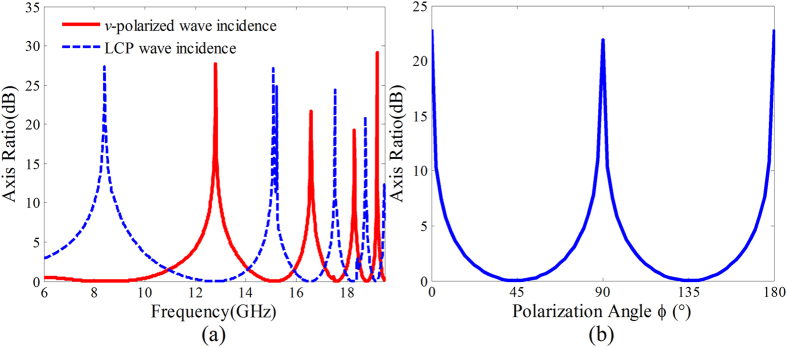
Axis ratio of the transmitted waves. (**a**) The axis ratio versus frequency of the transmitted waves for *v*-polarized and LCP waves normal incidence onto the microwave birefringent metamaterial. (**b**) The axis ratio of the transmitted wave versus the polarization angle *φ* of the incident LP wave at the frequency *f* = 8.01 GHz.

Due to the nonlinearly dispersive characteristic of the refractive index coefficients *n*_*x*_ and *n*_*y*_, the phase difference *φ*_*diff*_ between the *x*- and *y*-directions is nonlinearly dispersive. Thus the full-polarization states on the zero longitude line of the poincaré sphere can be achieved under *v*-(*u*-) polarized wave and CP wave incidence by varying the frequency. Twice full-polarization-state conversions can be fulfilled because the change of the phase difference is greater than 4π over the frequency range 6–20 GHz. Under *v-*(*u-*)-polarized wave normal incidence, the transmission coefficients in *x*- and *y*- directions approximately have the same amplitude. The polarization state of the transmitted wave changes with the changing of the phase difference *φ*_*diff*_. Specifically, the transmitted wave is LCP as *φ*_*diff*_ = π/2, *u*-polarized as *φ*_*diff*_ = π, RCP as *φ*_*diff*_ = 3π/2, and *v*-polarized as *φ*_*diff*_ = 2π. Under CP wave normal incidence, the transmittivities in *x*- and *y*-directions have the same value 0.5 due to the inherent near unity transmissions in the two directions. The polarization state of the transmitted wave can be modulated by varying the phase difference *φ*_*diff*_. To validate the polarization of the transmitted waves, the axis ratio of the transmitted wave versus frequency under *v*-polarized and LCP waves normal incidence were calculated from the simulated transmission coefficients. The calculated results given in Fig. 8(a) indicate that twice full-polarization-state conversions are fulfilled in the frequency ranging from 6 GHz to 20 GHz. In addition, the polarization state changes faster and faster with increasing frequency due to the nonlinearly dispersive phase difference.

### Full-polarization-state conversions achievement at a fixed frequency by varying the polarization angle of the LP incidence wave

At the frequencies *f* = 8.01, 15.01, 17.62 and 18.95 GHz, the phase differences *φ*_*diff*_ = ±π/2. The polarization state of the transmitted wave is determined by the assignation of the transmittivities in *x*- and *y*-directions. In detail, the transmitted wave is CP as the transmittivities in *x*- and *y*-directions have the same value, LP as the transmittivity in one direction is zero, elliptically polarized (EP) as the transmittivities in *x*- and *y*-directions have different values. The calculated axis ratio versus the polarization angle *φ* of the incident LP wave at frequency *f* = 8.01 GHz given in Fig. 8(b) indicates that the full-polarization state conversions can be achieved while the polarization angle is changed from 0° to 180°.

## Experimental Verification

**Figure 9:**
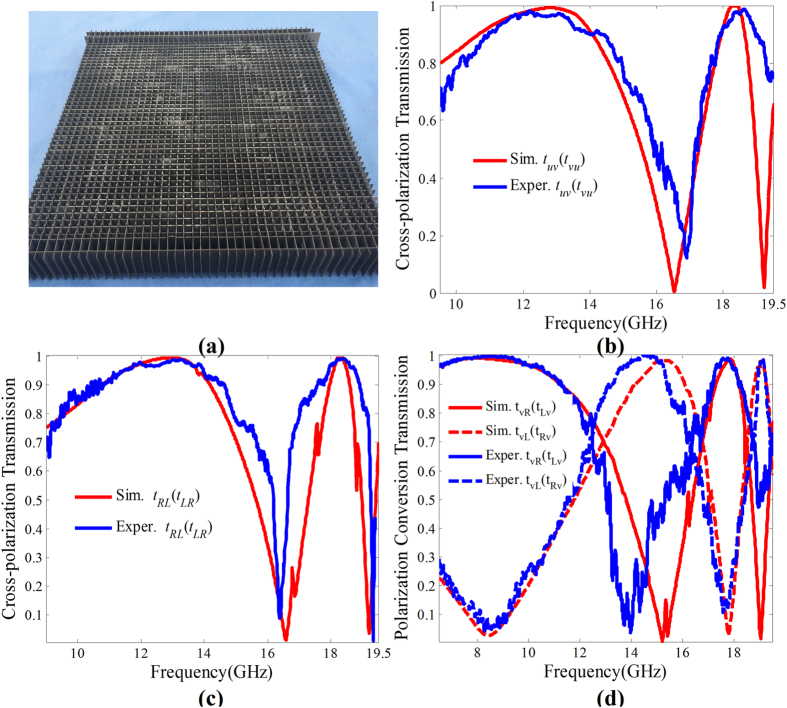
Experimental verifications of the CTC, LTC(CTL) and LTL polarization conversion transmissions for the microwave birefringent metamaterial. (**a**) The photograph of the fabricated microwave birefringent metamaterial sample, and the simulated and experimental (**b**) Cross-polarization transmissions under *v*-(*u*-)polarized wave normal incidence, (**c**) Cross-polarization transmissions under CP wave normal incidence, (**d**) LTC(CTL) polarization conversion transmissions.

The two different fishbone structures were fabricated using the print circuit board (PCB) technique. The final microwave birefringent metamaterial sample shown in Fig. 9(a) was achieved by crossly arranging the two fishbone structures. The amplitudes and phases of the co- and cross-polarization transmission coefficients were measured, respectively, for *y*-, *x*-, *v*- and *u*-polarized waves normal incidence, i.e. *t*_*yy*(*xx*)_, *t*_*yx*(*xy*)_, *t*_*vv*(*uu*)_, *t*_*vu*(*uv*)_, *ϕ*_*yy*(*xx*)_, *ϕ*_*yx*(*xy*)_, *ϕ*_*vv*(*uu*)_, and *ϕ*_*vu*(*uv*)_. Then the CTC cross-polarization transmission coefficients can be derived by the expressions,


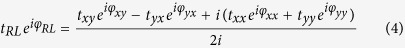



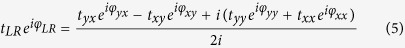


where *t*_*RL*(*LR*)_ and *φ*_*RL*(*LR*)_ are amplitude and phase, respectively. The LTC polarization conversion transmission coefficients under *v*-polarized wave normal incidence can be calculated using expressions (6) and (7),


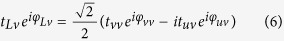



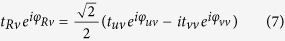


where *t*_*Lv*(*Rv*)_ and *φ*_*Lv*(*Rv*)_ are amplitude and phase, respectively.

To verify the polarization conversions of the microwave birefringent metamaterial, the simulated LTL and CTC cross-polarization transmissions, and LTC(CTL) polarization-conversion transmissions together with the experimental results are given in Fig. 9(b–d). It is found that the experimental results are well consistent with the simulated results. Both the experimental and simulated results indicate that the polarization converter can achieve dual-band, high-efficiency cross-polarization transmissions under *v*-(*u*-) polarized and CP wave normal incidence, and LTC (CTL) polarization conversion transmissions under *v*-(*u*-) polarized and CP wave normal incidence. In addition, the corresponding operating frequencies are in good accordance with the theoretical design.

## Conclusions

In conclusion, the SSPP mediated by the metallic blade structure is studied by the dispersion relationship. The transmission based on SSPP coupling is proposed and enhanced by improving the matching of the propagation constants between the SSPP and space wave using the fishbone structures. A birefringent metamaterial was designed by two different fishbone structures in two orthogonal directions with nonlinearly dispersive phase difference between them. The full-polarization-state conversions are fulfilled twice by varying the frequency under LP or CP wave incidence, as well as by varying the polarization angle of the incident LP wave at same frequencies. The dual-band, high-efficiency CTC, CTL (LTC) and LTL polarization conversion transmissions have been convincingly demonstrated by both the simulations and experiments.

## Methods

### Simulations

Electromagnetic simulations are performed using a commercially available software package, CST Microwave Studio. The dispersion relations are calculated using the Eigen-mode solver with periodic boundary conditions along the *x, y* and *z* directions. The amplitudes and phases of the transmission parameters are simulated using the Frequency domain solver and the electric field distributions are monitored simultaneously. In the simulation, unit cell boundary conditions in the *x* and *y* directions are used, and open boundary conditions in the *z* direction.

### Fabrication

The metallic fishbone structures are fabricated using the standard PCB photolithography. The commercial F4B dielectric substrates are employed as the dielectric layers and the 17-μm-thick copper films as the metal parts. The metallic fishbone structures in *yoz* and *xoz* planes were crossly arranged to obtain the final microwave birefringent metamaterial sample.

### Measurements

The experimental measurements of the polarization conversion transmissions are performed in a microwave anechoic chamber. In the measurement, a pair of horn antennas with standard gain faced upon each other. One works as a transmitter and the other as a receiver. The polarization of the transmitted wave or the received wave can be manipulated via rotating the horn antennas. The microwave birefringent metamaterial sample placed at the middle of the two horn antennas. The amplitudes and phases of the co- and cross-polarization transmissions for *y*-, *x*-, *v*- and *u*-polarized wave normal incidence were measured, respectively. Then the CTC, LTC (CTL) and LTL polarization conversion transmissions were derived.

## Additional Information

**How to cite this article**: Li, Y. *et al*. Microwave birefringent metamaterials for polarization conversion based on spoof surface plasmon polariton modes. *Sci. Rep.* **6**, 34518; doi: 10.1038/srep34518 (2016).
